# The Results of the Use of Ahmed Valve in Refractory Glaucoma Surgery

**DOI:** 10.5005/jp-journals-10008-1191

**Published:** 2016-02-02

**Authors:** Mukharram Mukhtaramovich Bikbov, Ilnur Ildarovich Khusnitdinov

**Affiliations:** Chief, Department of Management, Ufa Eye Research Institute Bashkortostan, Russian Federation, Russia; Head, Department of Microsurgery II, Ufa Eye Research Institute Bashkortostan, Russian Federation, Russia

**Keywords:** Ahmed glaucoma valve, Antimetabolites, Anti-VEGF drugs, Postoperative complications, Refractory glaucoma.

## Abstract

The treatment of refractory glaucoma (RG) is challenging. The commonly adopted strategy in RG treatment is a glaucoma drainage device (GDD) implantation, which despite its radical nature may not always provide the desired intraocular pressure (IOP) levels for a long term. This review is based on the scientific literature on Ahmed glaucoma valve (AGV) implantation for refractory glaucoma. The technique of AGV implantation is described and data for both the types, FP7 and FP8 performance are presented. The outcome with adjunct antimetabolite and anti-VEGF drugs are also highlighted. An insight is given about experimental and histological examinations of the filtering bleb encapsulation. The article also describes various complications and measures to prevent them.

**How to cite this article:** Bikbov MM, Khusnitdinov II. The Results of the Use of Ahmed Valve in Refractory Glaucoma Surgery. J Curr Glaucoma Pract 2015;9(3):86-91.

## INTRODUCTION

Glaucoma is the leading cause of irreversible blindness worldwide.^1^ The actual problem is the treatment of glaucoma refractory to medical treatment, laser and filtering operations. Mostly, refractory glaucoma patients include those with a previous failed trabeculectomy, neovascular glaucoma (NVG), uveal, aphakic and pseudo-phakic glaucoma.^2-4^

Common surgical treatments for refractory glaucoma are fistulizing operations and glaucoma drainage device (GDD) implantation. Perhaps, the most common GDD used in refractory glaucoma (RG) surgery is Ahmed glaucoma valve (AGV), which was developed in 1993. Among fistulizing surgeries, trabeculectomy remains the gold standard for most cases of glaucoma worldwide;^5^ but, in cases of RG, it retains its effectiveness in the short term and over time often additional medications or surgeries are required.^6-11^ Furthermore, trabeculectomy is associated with a high incidence of early and late postoperative complications.^12^ Drainage operations, the principle of which was proposed in 1912 by Zorab,^13^ have been used since long for RG surgery. Valved and valveless drainage devices available are Molteno (Molteno Ophthalmic Ltd., New Zealand), Baerveldt (Advanced Medical Optics, USA), Ahmed (New World Medical Inc., Rancho Cucamonga, CA, USA) and Krupin (Hood Laboratories, USA). Ahmed and Baerveldt implants are the most frequently used among these drainage devices.^14^

## EFFECTIVENESS OF AHMED GLAUCOMA VALVE

The AGV was launched in 1993 as the first GDD with a unidirectional valve mechanism contributing to the prevention of postoperative hypotension.^15^ Currently, there are two models of AGV which differ in their surface areas: FP8 (96 mm^2^) is used in children and FP7 (184 mm^2^) is usually used in adults.

It has been hypothesized that large drainage devices increase the encapsulation area and, thus, provide a high degree of intraocular pressure (IOP) drop.^16^ In a prospective study by Lloyd et al,^17^ a comparison of Baerveldt valve with sizes 350 and 500 mm^2^ showed no difference in efficacy and visual results. Kang and Kee^18^ claimed that there is an upper limit of increase in the drainage surface area when a beneficial effect on IOP is not marked. In their study, Koh et al^19^ did not observe any significant difference in effectiveness of FP8 and FP7 implantation. At a 3-year follow-up, the efficacy was 79.2% for FP8 and 72.7% for FP7, which was comparable with other studies.^19^

The FP7 type of AGV is preferred for use in the eyes of adult patients. However, the implantation of AGV FP7 model is a challenging task in patients with small anteroposterior dimensions of eyes, or if there is scarring of the conjunctiva, due to previous surgeries or inflammatory diseases of the eye. In such eyes, a large implant may lead to various complications, such as extrusion, discomfort and surgical wound dehiscence.^20^ For such cases, the FP8 model may be preferable.

According to different authors, the AGV implantation is considered an effective treatment option for patients with RG and the success varies in a wide range―from 43 to 83.6%.^14,15,21-25^

Coleman et al determined the efficacy of AGV implantation in 78% of cases at 12 months after surgery.^15^ Hu et al studied patients from Korea, with 6-month observation period and established efficacy in 80% of cases.^11^ Das et al reported that the efficacy of AGV implantation in India 12 months postoperatively was 53% which reduced to 43% in 2 years.^26^ Shah et al analyzed the results of AGV implantation in adult Arab population with RG in Oman and reported 12% absolute and 78% relative success of IOP compensation at 6 months.^27^ Ishida and Netland^28^ reported that African American patients were more often faced with implant failure, especially in NVG and previously operated glaucoma. In general, according to the literature, the efficacy of the tubular drainage devices is reduced by about 10% within 1 year, and, by 5 years of follow-up, implants operate effectively in about 50% of cases only.^29,30^

### Encapsulation of the Filtering Bleb

The efficacy of a bypass glaucoma surgery depends largely on formation of a semipermeable capsule around the drainage device body, which determines the rate of intraocular fluid resorption and, thus, the degree of IOP decrease.^16^ Formation of an encapsulated cyst of the filtering bleb is related to late complications of the AGV implant. According to different authors, the occurrence of encapsulated cyst formation varies from 5 to 30%, depending on the period of observation and patient selection. According to a retrospective analysis by Lima et al, such cysts after AGV implantation were formed in 14.7% of cases.^23^ Lai et al observed 65 eyes with an AGV implant and noted encapsulated filtering blebs in 16 cases (16/65; 24.6%).^30^

Causes of excessive scarring and encapsulation of the filtering bleb are not completely studied. It is believed that the formation of the encapsulated filtering bleb depends largely on the properties of an implant, namely, its size, shape, surface of the biomaterial, which leads to adhesion and proliferation of fibroblasts.^31^ As per the reported literature, the capsule wall in cases of unsuccessful AGV implant is macroscopically thicker than the wall of an encapsulated filtering bleb after trabeculectomy. However, histologically there is no difference between them.^32^ It is known that the wall of the encapsulated filtering bleb after AGV implantation is divided macroscopically and histologically into two layers. The outer surface is roughly vascularized, while the inner surface is smooth due to the densely packed compressed collagen fibers and activated myofibroblasts.^33^ Recent studies of a filtering bleb carried out using the optical coherence tomography of the anterior segment (ASOCT) of the eye found out that after a successful, functional AGV implantation, the wall of the filtering bleb was much thinner compared to a dysfunctional implant.^34^

Lee et al conducted a histological examination of the fibrous capsule around AGV implanted with amnio-tic membrane in the rabbit eyes. A fibrous capsule consisting of compact collagen fibers with minimal vascularization was seen in the control group. In contrast, the study group had a thinner myofibroblasts layer with disorganized collagen fibers in the fibrous capsule. The authors established that the use of an additional amniotic membrane may reduce the risk of encapsulation by forming a loose thin capsule around the AGV.^35^

### Antimetabolites and Anti-VEGF Drugs

Antimetabolite application can significantly inhibit fibrosis, and is widely used in drainage and fistulizing glaucoma surgeries.^36-38^ However, several authors have not reported any effectiveness of mitomycin C (MMC) with AGV implantation, neither in the short nor in medium term follow-up.^39,40^ Recently, a new method has been described to prevent the formation of the encapsulated bleb in patients with RG after AGV implant. According to this new technique, the valve plate is wrapped in a thin layer of tissue soaked in MMC (0.25-0.33 mg/ml), and then placed over the implantation area with subsequent removal of tissue after 2 to 5 minutes, and profuse washing out of the surgical field with balanced salt solution (BSS). Efficacy and encapsulation of the filtering bleb according to this new technique were respectively 89.5 and 2.6%, while in the group with the traditional method they were 70.7 and 19.5% respectively.^41^

Alvarado et al used a tubular implant with additional antimetabolite application (both intraoperatively and postoperatively) as weekly injections for 5 weeks increases the efficacy of the surgery. It is associated with a low probability of hypertensive phase (which typically occurs between 3 weeks and 6 months postoperatively) and fewer postoperative complications.^36^ However, postoperative reinterventions are inconvenient and risky for patients due to a possibility of a secondary infection by micro perforation of the filtering bleb after needling.

There is a flagrant necessity to create drug delivery systems that can be installed intraoperatively to deliver antimetabolites during the wound-healing phase after the implantation of drainage devices. Schoenberg et al^42^ conducted a study of two drug delivery systems integrated with the AGV, namely, a nonbiodegradable poly (2-hydroxyethyl methacrylate) system with MMC and biodegradable poly lactic-co-glycolic acid system with 5-fluorouracil (5-FU). The authors reported a safe concentration and pattern of the antimetabolite release, reduction in thickness of the filtering bleb and fibrous capsule.

Eid et al^43^ noted a positive role of intravitreal bevaci-zumab (IVB) in improving the effectiveness of bypass surgery of NVG. According to some studies, IVB with AGV implantation in patients with NVG reduces a number of hemorrhagic complications, but the valve efficacy remains the same; IOP values have not been fundamentally different.^44,45^ Zhang et al investigated the efficacy of IVB given prior to AGV implantation in 35 patients (35 eyes) with NVG. The efficacy was 82.9, 74.1 and 71.0% in terms of 12, 24 and 36 months respectively.^46^ In another study, the authors conducted a comparative study between the two groups of patients with NVG, which had IVB injection before AGV and without it. They reported the efficacy at 12 months after surgery to be 84.0% (IVB and AGV) and 64.3% (AGV) and, after 18 months, 80.0 and 53.6% respectively. Preoperative administration of IVB significantly reduces the hyphema occurrence. Iris neovascularization regression occurred 2 to 10 days after IVB injection.^47^

### Implantation Technique and Complications

Pakravan et al compared the effectiveness of the AGV implantation in the upper and lower sectors. Their study showed that the effectiveness was the same, but complications (cosmetic discomfort, tube eruption, endophthalmitis, diplopia) were more marked in the lower sector.^48^ A new technique of sutureless fixation of AGV using cyanoacrylate adhesive was described in 17 patients (17 eyes) with RG. The AGV efficacy was noted to be 82.2% and there was no eruption or dislocation of the valve tube. Transient hypertension, hyphema, early postoperative hypotension were seen in four cases, tube blockage with vitreous body was seen in two cases: in case, it was broken by Nd:YAG laser, while in the second case, an anterior vitrectomy was done.^49^ However, a simple valve implantation regardless of the sector of the surgery often causes severe complications, such as choroid detachment, shallow anterior chamber, transscleral eruption of the tube, valve dislocation, hypotension, diplopia, decompensation of the cornea, cataract, intraocular hemorrhage, in some cases, retinal detachment, endophthalmitis.^50-52^

According to the literature sources, a choroidal detachment after valve implantation may develop in 8 to 22% of cases.^15,53^ Hypotension due to choroidal effusion has a significant damaging effect on the eye and may lead to loss of vision, thus, reducing the efficacy of the surgery itself. Even after a successful implantation of a AGV, many patients may have complications in the late postoperative period, due to the proximity of the tube to the endothelial layer,^54,55^ or due to contact of AGV tube with the cornea, which, according to Topouzis et al, occurs in 5% of cases.^56^

Lee et al investigated the rate of change in endothelial cells number after AGV implantation for 24 months. The average number of endothelial cell lost after AGV implantation was 5.8% within 1 month, 11.5% after 6 months, 15.3% after 12 months, 16.6% after 18 months and 18.6% after 24 months. The greatest loss of endothelial cells was 22.6% and was observed in the area of the valve tube, while in the central area of the cornea the loss was only 15.4%, even 24 months after the surgery.^57^ The literature also reports a few cases of AGV tube retraction, which may develop in the long term. According to Topouzis et al, the occurrence of this complication was seen in one in 31 patients,^56^ whereas Budenz et al reported it in one in 413 patients.^14^ Movement of the tube may occur due to loosening of the nonabsorbable suture, its gradual sagging and/or marked proliferation of fibroblasts around the valve plate.^58^ One of the known complications of AGV implantation is erosion of the tube through the sclera and conjunctiva. Previously, transcorneal dislocation of the AGV tube was described.^59^ Chances of tube erosion through the conjunctiva can be reduced by coating the tube with either of the graft materials *viz* sclera,^60^ scleromeninx,^61^ fascia,^62^ pericardium^63^ and autologous sclera.^64^ In one study, a comparative analysis of application of various coating materials for GDD tube, donor sclera, pericardium and scleromeninx, showed no dependence of the valve tube erosion on the graft tissue.^65^ Furthermore, suturing for graft fixation might introduce an infection and cause subsequent melting and rejection of the graft.^66^ Fixation of the graft using fibrin glue has been reported to be safer and more efficient as compared to suture fixation.^67^

Inflammatory and/or immunologically mediated melting of self-tissue or the donor graft and subsequent mechanical damage by the underlying valve tube of the overlying conjunctiva lies at the heart of protruding mechanism of the valve tube erosion.^68^ The bare tube is coated with self-conjunctiva, donor sclera, amniotic membrane, buccal mucosa, etc.^69^ However, if these techniques are unsuccessful, then the valve should be explanted. Hu et al described four cases of valve explantation 1.5 to 9 months after the surgery due to the conjunctival erosion in three cases, and constant diplopia in one case.^70^ Sibayan and Latina described a technique by covering a fistula formed at the site of the silicone tube in the cornea and sclera, with a treated pericardium after valve explantation.^71^ The followers of this technique, Yoo et al observed their patients for 26 months and reported no complications, such as rejection or melting of the pericardium, direct filtration, wound infection and endophthalmitis.^72^

Endophthalmitis is a rare complication after GDD implantation and occurs in 0.8 to 6.3% of cases.^17,73,74^ In particular, Morad et al reported three cases of endo-phthalmitis after AGV implantation, two of their cases were associated with tube erosion and subsequent infection. Explantation of the graft, vitrectomy and intravitreal injection of antibiotics resulted in inflammation relief.^74^

Analysis of the published data suggests that AGV implantation has proved itself as an effective surgical modality for refractory glaucoma. It is safe to conclude that the number of successful AGV implants outnumbers the complicated or failed cases.
